# Unintentional sulfonylurea toxicity due to a drug–drug interaction: a case report

**DOI:** 10.1186/s13104-018-3404-8

**Published:** 2018-05-21

**Authors:** Keith Gunaratne, Emily Austin, Peter E. Wu

**Affiliations:** 1 Department of Medicine, University of Toronto, Toronto, ON Canada; 2 Division of Emergency Medicine, University of Toronto, Toronto, ON Canada; 3 Division of Clinical Pharmacology & Toxicology, University of Toronto, Toronto, ON Canada; 4 Division of General Internal Medicine, University Health Network, 200 Elizabeth St. 14EN206, Toronto, ON M5G 2C4 Canada

**Keywords:** Education, Toxicology, Pharmacology, Drug-interaction

## Abstract

**Background:**

Sulfonylureas are widely used for type 2 diabetes mellitus, but these medications carry a risk of hypoglycemia. Drug–drug interactions that inhibit sulfonylurea metabolism and thus increase systemic exposure can cause unintentional sulfonylurea toxicity.

**Case presentation:**

A 56-year-old man presented with severe, recurrent hypoglycemia. He had a history of type 2 diabetes mellitus and was taking the sulfonylurea gliclazide with no prior episodes of hypoglycemia. The onset of his hypoglycemia occurred within days after starting voriconazole and subsequently fluconazole for a fungal pneumonia. Unintentional sulfonylurea toxicity developed due to an adverse drug–drug interaction between gliclazide and these antifungals. Azole antifungals inhibit the metabolism of sulfonylureas resulting in increased systemic exposure and consequent toxicity. After the diagnosis of sulfonylurea toxicity was recognized, the patient was treated initially with dextrose and then administered octreotide to prevent recurrent hypoglycemia. He was successfully managed, his hypoglycemic episodes resolved, and his medications were adjusted to avoid any further adverse interactions.

**Conclusions:**

Adverse drug–drug interactions continue to pose challenges to clinicians. Both individual vigilance and system wide strategies are needed to prevent and mitigate consequences. This case highlights an important drug–drug interaction and reviews the presentation, management and antidotal therapy of sulfonylurea toxicity.

## Background

Sulfonylureas are commonly used for type 2 diabetes mellitus. These compounds stimulate glucose-independent endogenous insulin release [1] and carry an inherent risk of hypoglycemia, particularly in the setting of intentional overdose or due to adverse drug interactions. Sulfonylureas are extensively metabolized by the hepatic cytochrome P450 (CYP) 2C9 isoform and medications that inhibit this enzyme can result in impaired sulfonylurea metabolism, increased exposure and consequent toxicity [2].

We present a case of severe, recurrent hypoglycemia in a patient taking the sulfonylurea gliclazide who was prescribed voriconazole followed by fluconazole, two inhibitors of the hepatic CYP2C9 enzyme. Once recognized, this unintentional overdose was successfully managed initially with dextrose, and then octreotide.

## Case presentation

A 56-year-old man presented to the emergency department with new onset hypoglycemia. He had a history of type 2 diabetes treated with metformin (2500 mg orally daily in divided doses), gliclazide modified release (90 mg orally daily), and a multiple daily injection insulin regimen (insulin glargine 18 units subcutaneously at night and insulin lispro 6 units subcutaneously with meals). He was adherent with all medications and had never developed hypoglycemia. Past medical history was notable for a bone marrow transplantation for myelofibrosis 8 months prior and long-term treatment with prednisone (5 mg orally daily), resulting in an immunocompromised state.

One week before presentation, he was evaluated in clinic for persistent cough. Findings on a Computed Tomography scan of the thorax were suspicious for fungal pneumonia, and he was empirically treated with voriconazole (200 mg orally twice daily). He was reassessed in clinic 6 days later reporting lethargy, visual disturbances, and hallucinations. Voriconazole intolerance was suspected, as visual disturbances and hallucinations are well described side effects [3], and his antifungal was switched to fluconazole (480 mg orally daily). Incidentally, after starting antifungal therapy, he had not required any short-acting insulin due to glucose readings of 54–72 mg/dL (3.0–4.0 mmol/L) (reference range 72–126 mg/dL; 4.0–7.0 mmol/L).

The day after initiating fluconazole, he presented to a follow-up appointment at 1800 h weak and tremulous. His capillary glucose was 31 mg/dL (1.7 mmol/L). He was given orange juice, felt better, and sent to the emergency department, where a repeat measurement at 2000 h was 34 mg/dL (1.9 mmol/L). He consumed more juice and a sandwich, his symptoms improved and subsequent hourly glucose measurements ranged between 68 and 106 mg/dL (3.8–5.9 mmol/L). At 0100 h his glucose was 106 mg/dL (5.9 mmol/L) and he fell asleep.

At 0430 h, he was woken for reassessment. He was confused, lethargic and glucose was 25 mg/dL (1.4 mmol/L). The recurrent nature of his hypoglycemia in the setting of recent azole antifungal co-prescription was concerning for gliclazide toxicity due to an adverse drug interaction.

He was treated with a 50 cc bolus of 50% dextrose followed by a 100 cc per hour infusion of 10% dextrose. In consultation with the regional poison centre, he was given octreotide 75 μg subcutaneously for suspected sulfonylurea toxicity and the intravenous dextrose was discontinued as his blood sugar normalized. One further dose of octreotide was administered 6-h later and his hypoglycemic episodes abated with no recurrence during hospitalization. His gliclazide was held and his medical records were updated to document this interaction.

## Discussion and conclusions

Sulfonylureas stimulate insulin release independent of plasma glucose concentration, thereby creating a risk for hypoglycemia. Hypoglycemia can occur due to accidental ingestion, intentional overdose [4], or as a result of adverse drug–drug interactions [5]. Sulfonylureas are metabolized by CYP2C9 and the most common drug–drug interaction causing toxicity is inhibition of this enzyme [2]. The azole antifungals voriconazole and fluconazole are examples of CYP2C9 inhibitors and there are many commonly prescribed drugs that can inhibit or induce this enzyme (Table 1) [1, 2, 5, 6]. One retrospective study of 3884 patients on sulfonylureas revealed that 20% were co-prescribed a CYP2C9 inhibitor and were more likely (12.8% vs. 8.9%) to have a fasting glucose below 72 mg/dL (4 mmol/L) [5]. Another retrospective population-based study of 909 elderly patients on sulfonylureas admitted to hospital with hypoglycemia were more than six times as likely to have been prescribed co-trimoxazole (a CYP2C9 inhibitor) in the previous week than matched controls [7].

**Table 1:** Common medications that can interact with sulfonylureas [1, 2, 5, 6]

Drug	Mechanism	Effect
Fluconazole, ketoconazole, voriconazole, amiodarone, co-trimoxazole (sulfamethoxazole component), fluoxetine, fluvoxamine, metronidazole	Inhibition of CYP2C9	Hypoglycemia
Carbamazepine, phenobarbital, rifampin, St. John’s wort, dexamethasone	Induction of CYP2C9	Reduced efficacy (hyperglycemia)

The management goals of sulfonylurea toxicity are to restore and maintain euglycemia. The initial hypoglycemic episode will require an intravenous (IV) dextrose bolus, followed by complex carbohydrates; however, ongoing IV dextrose use can complicate recovery and should be avoided. Glucose independently stimulates insulin release, thus ongoing administration of dextrose in the setting of sulfonylurea toxicity will perpetuate a dangerous cycle of recurrent hypoglycemia [8]. Octreotide is a long-acting somatostatin analog that prevents the secretion of insulin downstream of sulfonylurea action. It is recommended as part of sulfonylurea toxicity management to prevent recurrent hypoglycemia [8, 9]. A prospective randomized study of 40 emergency department patients with sulfonylurea-induced hypoglycemia demonstrated higher glucose levels and fewer episodes of recurrent hypoglycemia over an 8-h period after treatment with octreotide 75 μg subcutaneously compared to placebo [10].

There is limited published data to guide dosing protocols for octreotide. A recommended strategy is octreotide 50–100 μg subcutaneously every 6–12 h as needed [8]. Patients require observation for a minimum of 12-h after the last octreotide dose to ensure no recurrence of hypoglycemia [8]. Octreotide is well tolerated and only minor adverse events have been associated with its use [4].

This case also highlights the importance of vigilance for drug–drug interactions, and the need for better systems to aid in identifying clinically significant interactions. Drug–drug interactions are innumerable and it is unreasonable to expect clinicians to recall every potential interaction. However, whenever new medications are started it is sensible for clinicians to search for interactions between the new drug and any high-risk medications with narrow therapeutic indices (e.g. oral antihyperglycemics, anticoagulants, opioids). Collaboration with pharmacists and iterative medication review would assist in identifying adverse drug interactions.

Sulfonylureas carry an inherent risk of hypoglycemia due to a mechanism of glucose-independent insulin release. Unintentional toxicity can occur if sulfonylurea metabolism is impaired by a co-prescribed medication that inhibits CYP2C9. In this case, the addition of azole antifungals was the culprit. Sulfonylurea toxicity is readily treated if recognized quickly. Management includes judicious use of intravenous dextrose, ingestion of complex carbohydrates and administration of octreotide to prevent recurrent hypoglycemia. Drug–drug interactions remain a common and preventable source of harm associated with medical care, both individual vigilance and system wide changes are necessary to prevent and mitigate these adverse drug events.

## Abbreviation

CYP: cytochrome P450
